# Effective combination of human bone marrow mesenchymal stem cells and minocycline in experimental autoimmune encephalomyelitis mice

**DOI:** 10.1186/scrt228

**Published:** 2013-07-05

**Authors:** Yun Hou, Chung Heon Ryu, Kwang Ywel Park, Seong Muk Kim, Chang Hyun Jeong, Sin-Soo Jeun

**Affiliations:** 1 Department of Biomedical Science, College of Medicine, The Catholic University of Korea, 505 Banpo-daero, Seoul, Seocho-gu, Seoul 137-701, Korea; 2 Postech-Catholic Biomedical Engineering Institute, Seoul St. Mary’s Hospital, The Catholic University of Korea,505 Banpo-daero, Seocho-gu, Seoul 137-701, Korea; 3 Department of Neurosurgery, Seoul St. Mary’s Hospital, The Catholic University of Korea,505 Banpo-daero, Seocho-gu, Seoul 137-701, Korea

**Keywords:** hBM-MSCs, Minocycline, Demyelination, Neuroprotection, EAE, MS

## Abstract

**Introduction:**

Multiple sclerosis (MS) is the most common inflammatory demyelinating disorder of the central nervous system (CNS). Minocycline ameliorates the clinical severity of MS and exhibits antiinflammatory, neuroprotective activities, and good tolerance for long-term use, whereas it is toxic to the CNS. Recently, the immunomodulation and neuroprotection capabilities of human bone marrow mesenchymal stem cells (hBM-MSCs) were shown in experimental autoimmune encephalomyelitis (EAE). In this study, we evaluated whether the combination of hBM-MSCs and a low-dose minocycline could produce beneficial effects in EAE mice.

**Methods:**

The sensitivity of hBM-MSCs to minocycline was determined by an established cell-viability assay. Minocycline-treated hBM-MSCs were also characterized with flow cytometry by using MSC surface markers and analyzed for their multiple differentiation capacities. EAE was induced in C57BL/6 mice by using immunization with MOG35-55. Immunopathology assays were used to detect the inflammatory cells, demyelination, and neuroprotection. Interferon gamma (IFN-γ)/tumor necrosis factor alpha (TNF-α) and interleukin-4 (IL-4)/interleukin-10 (IL-10), the hallmark cytokines that direct Th1 and Th2 development, were detected with enzyme-linked immunosorbent assay (ELISA). terminal dUTP nick-end labeling (TUNEL) staining was performed to elucidate the cell apoptosis in the spinal cords of EAE mice.

**Results:**

Minocycline did not affect the viability, surface phenotypes, or differentiation capacity of hBM-MSCs, while minocycline affected the viability of astrocytes at a high dose. *In vivo* efficacy experiments showed that combined treatment, compared to the use of minocycline or hBM-MSCs alone, resulted in a significant reduction in clinical scores, along with attenuation of inflammation, demyelination, and neurodegeneration. Moreover, the combined treatment with hBM-MSCs and minocycline enhanced the immunomodulatory effects, which suppressed proinflammatory cytokines (IFN-γ, TNF-α) and conversely increased anti-inflammatory cytokines (IL-4, IL-10). In addition, TUNEL staining also demonstrated a significant decrease of the number of apoptotic cells in the combined treatment compared with either treatment alone.

**Conclusions:**

The combination of hBM-MSCs and minocycline provides a novel experimental protocol to enhance the therapeutic effects in MS.

## Introduction

Multiple sclerosis (MS) is an inflammatory demyelinating disease of the central nervous system (CNS). The characteristics of MS include multifocal perivascular mononuclear cell infiltrates in the CNS, demyelination, and neuronal loss. To date, several therapeutic strategies have been studied in experimental autoimmune encephalomyelitis (EAE) mice. However, further improvement in MS therapeutics is necessary, with a focus on preventing the infiltration of inflammatory cells into the CNS and/or preventing demyelination and apoptotic cell death.

Minocycline is a semisynthetic tetracycline analogue suitable for treatment of CNS disorders because it is capable of penetrating the blood–brain barrier and has antiinflammatory and antiapoptotic activities. It is effective in delaying progression in numerous neurodegenerative diseases. The use of minocycline in EAE and MS can attenuate disease activity [1,2] and reduce magnetic resonance imaging (MRI)-detected gadolinium enhancements within 2 months of treatment [3]. In addition, minocycline exerts neuroprotective effects in EAE by protecting axons from demyelination; attenuating neuronal death [4]; and modulating immune differentiation from a Th1 toward a Th2 phenotype, thereby reducing T-cell infiltration into the spinal cord [2,5]. The pathogenic complexity and heterogeneity of MS makes combination therapy an attractive treatment strategy. Previous studies of suboptimal doses of minocycline combined with interferon-beta (IFN-β), methylprednisolone, or atorvastatin have demonstrated better outcomes in EAE than any of these drugs used [6-8]. However, minocycline can cause adverse effects, such as systemic lupus erythematosus and serum sickness [9,10], and is toxic to the CNS at high doses [11,12]. Therefore, it is necessary to find a combined therapy that requires a low dose of minocycline.

Human bone marrow mesenchymal stem cells (hBM-MSCs) have been viewed as a potential treatment for neurodegenerative diseases. They are easily obtained from human bone marrow and escape immune system surveillance because they possess cell-surface antigens that are poorly recognized by T cells [13], and facilitate engraftment. hBM-MSCs are able to suppress T-lymphocyte activation and proliferation, induce a Th2-polarized immune response, and promote endogenous repair, thus demonstrating immunomodulatory capacities both *in vitro* and *in vivo*[14-17]. In addition, previous studies have shown that on engraftment, hBM-MSCs selectively migrate and target damaged tissue, providing a feasible and practical way to combat inflammation, reduce demyelination, and protect neurons and axons in EAE [18-20].

In this study, we investigated whether the combination of hBM-MSCs and minocycline would produce beneficial effects in EAE mice. We demonstrate that the combination treatment delayed clinical onset; attenuated clinical severity, inflammation, and demyelination; and enhanced neuroprotection when compared with either treatment alone. Most important, this combination treatment enhanced immunomodulatory functions, suggesting that it has potential to improve the functional recovery of patients with MS.

## Materials and methods

### Cell culture and reagents

hBM-MSCs were purchased from (Lonza, Walkersville, IN, USA). Cells were thawed, and initiation of the culture process was performed according to the manufacturer’s instructions. Cells were plated in a culture dish and cultured with hBM-MSC basal medium supplemented with MSC growth supplement at 37°C in a humidified atmosphere containing 5% CO_2_. Astrocytes were obtained from the American Type Culture Collection (ATCC, Manassas, VA, USA). The cells were grown in Dulbecco modified Eagle medium (Invitrogen, Carlsbad, CA, USA) containing 10% fetal bovine serum. Minocycline was purchased from Sigma-Aldrich (St. Louis, MO, USA), dissolved in distilled water at 1 m*M* and filter-sterilized.

### Assessment of MSC viability and characterization to minocycline

hBM-MSCs or astrocytes were seeded in 24-well plates (8 × 10^3^) or 96-well plates (5 × 10^3^), respectively. Increasing amounts of minocycline were added to confirm minocycline hBM-MSC or astrocyte-specific cytotoxicity. Twenty-four hours after treatment, cell viability was analyzed with the (3-(4,5-dimethylthiazol-2-yl)-2,5-diphenyltetrazolium (MTT) assay (Sigma-Aldrich). Fluorescence-activated cell sorting (FACS) was performed to evaluate cell-surface markers. hBM-MSCs treated with or without minocycline were trypsinized, washed with phosphate-buffered saline (PBS), and then incubated with phycoerythrin-conjugated mouse anti-human CD34, CD45, HLA-DR, CD73, CD90, and CD44 antibody (all from BD Bioscience, Franklin Lakes, NJ, USA). The differentiation of hBM-MSCs to adipogenic or osteogenic lineages was induced, as described previously, with or without minocycline [21]. After 3 to 4 weeks culture in induction medium with or without minocycline, the differentiated cells were fixed with 10% formaldehyde. Adipocytes were detected by staining the lipid droplets in the cell by using 0.3% Oil Red O staining for 10 minutes. Osteocytes were detected with calcium phosphate deposits by using 0.2% Alizarin Red S staining for 20 minutes.

### EAE induction and treatment

All animal protocols were approved by the Institutional Animal Care and Use Committee of the Catholic University Medical College. EAE was induced in C57BL/6 mice (female, 11 weeks old) by immunization with MOG35-55 (Hooke Labs, Lawrence, MA, USA). The mice were injected subcutaneously at two sites with a total of 200 μg of MOG35-55 emulsified in complete Freund adjuvant (CFA) containing 6 mg/ml of *Mycobacterium tuberculosis*. Two and 24 hours after the MOG35-55 injection, the mice received 100 ng pertussis toxin intraperitoneally. Paralysis as clinical evidence of EAE was assessed daily starting on day 5 after immunization, when all the mice were still clinically normal. Clinically, animals were scored as follows: 0, no clinical signs; 1, limp tail; 2, partial hind-leg paralysis; 3, complete hind-leg paralysis; 4, complete hind-leg paralysis and partial front-leg paralysis; and 5, moribund or dead. Mice were randomly divided into four groups: PBS (*n* = 10), hBM-MSCs (1.5 × 10^6^ cells in 100 μl PBS for each mouse, intravenous injection, *n* = 10), minocycline (10 mg/kg, intraperitoneal injection, *n* = 10), and combination of hBM-MSCs and minocycline (*n* = 10). All treatments started on day 7 after immunization. Minocycline was administered intraperitoneally for consecutive days until death.

### Immunohistopathology

Mice were killed on day 46 after immunization. Frozen sections were obtained from lumbar spinal cords and processed for hematoxylin and eosin (H&E) staining, Luxol Fast Blue (LFB) staining, and immunofluorescence staining to evaluate the presence of inflammatory cells, demyelination, neuronal loss, and activated gliocytes, according to standard protocols. For immunofluorescence, lumbar spinal cord sections were incubated at 4°C overnight with the following antibodies: monoclonal mouse anti-glial fibrillary acidic protein (GFAP; Millipore, Temecula, CA, USA), polyclonal rabbit anti-ionized calcium-binding adaptor molecule 1 (Iba1; Wako Pure Chemical Industries, Osaka, Japan), monoclonal mouse antineuronal nuclear antigen (NeuN; Chemicon International, Temecula, CA, USA), monoclonal mouse anti-CD4 (BD Biosciences Pharmingen, CA, USA), and polyclonal rabbit anti-mouse myelin basic protein (MBP; Millipore, Billerica, MA, USA). Antibody staining was visualized with anti-rabbit and anti-mouse Cy3-conjugated secondary antibodies (Jackson ImmunoResearch, West Grove, PA, USA). Specificity of immunoreactivity was confirmed by the absence of an immunohistochemical reaction in sections from which primary or secondary antibodies were omitted. In all sections, counterstaining of cell nuclei was carried out by incubating the sections with 4-6-diamidino-2-phenyindole (DAPI; Roche, Penzberg, Germany) for 10 minutes. All images were acquired by using an LSM 700 confocal microscope (Carl Zeiss, Oberkochen, Germany).

### Determination of serum cytokines with enzyme-linked immunosorbent assay

Serum was obtained from all animals of each treatment group on day 40 after immunization. ELISA was performed by using Quantikine immunoassay from R&D Systems (Madison, WI, USA). In brief, sera were incubated in the precoated 96-well plates for 4 hours at room temperature (RT). After three washes, conjugated antibody was added for 2 hours at RT, incubated in the substrate solution for 30 minutes, and the reaction was stopped by the addition of stop solution. The optical density of each well was determined by using a microplate reader at 450 nm.

### Determination of the apoptotic cell death with TUNEL assay

Apoptotic cells were visualized by using a terminal deoxynucleotidyl transferase dUTP nick-end labeling (TUNEL) assay kit (Roche, Basel, Switzerland) developed by using Cy3-conjugated streptavidin (Jackson ImmunoResearch Laboratories). In brief, slides were placed in the distilled water at 60°C for 2 hours, after washing with terminal deoxynucleotidyl transferase (TdT) labeling buffer, TUNEL reaction mixture was pipetted onto the sections, which were then incubated in a humidified chamber at 37°C for 1 hour. The reaction was stopped by adding terminating buffer. Counterstaining of cell nuclei was carried out by incubating the sections with DAPI for 10 minutes. In addition to characterization of the apoptotic cell, the sections were then double labeled with NeuN, GFAP, Ib-1, and CD4, respectively.

### Quantification and statistical analysis

Quantification was performed by an examiner blinded to the treatment status of each animal. Six to eight sections from each of three transverse lumbar spinal cords collected from each group were qualitatively analyzed. H&E and LFB stains were viewed with a Slide Scanner for Digital Pathology (SCN400; Leica, Wetzlar, Germany) under a × 200 objective lens, and immunofluorescence was viewed with a confocal microscope under a × 200 objective lens. All images were measured with MetaMorph software, version 7.5 (Molecular Devices, Sunnyvale, CA, USA). The average number of infiltrated cells, positive staining cell number, or fluorescence intensity was presented as the cell number or fluorescence intensity in the lesion sites under each photographed objective magnification. Lesion size was determined with quantitative histologic analysis of the LFB-counterstained spinal cord sections. The lesion size was presented as lesion area under photographed objective magnification. Data are presented as mean ± SEM. All statistical comparisons between the groups were examined by using one-way analysis of variance (ANOVA) with *post hoc* Bonferroni corrections. The *P* values <0.05 were considered statistically significant.

## Results

### Effects of minocycline on hBM-MSC viability, phenotype, and differentiation

**Figure 1:**
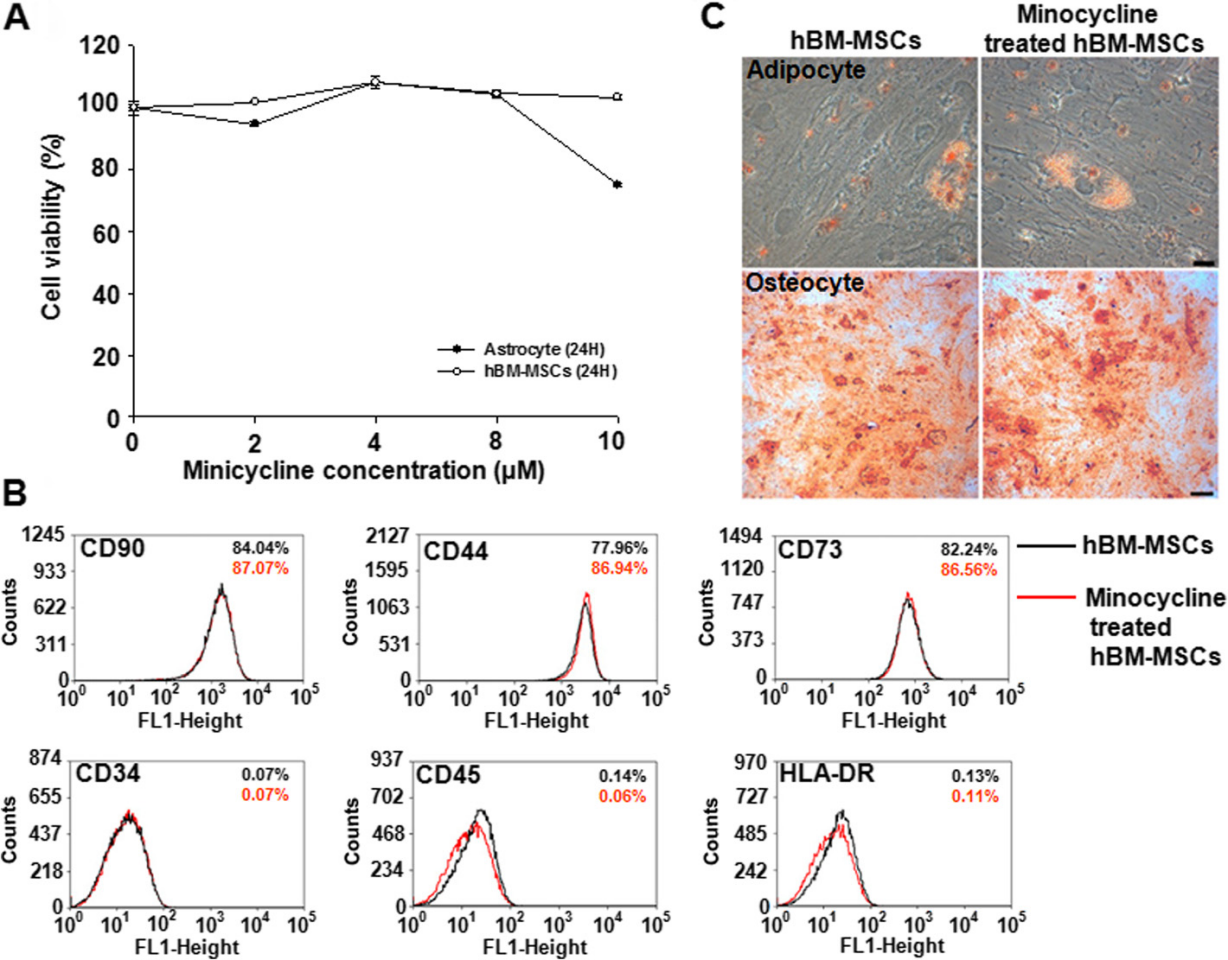
**Effects of minocycline on hBM-MSC stability. (A)** hBM-MSC and astrocyte viabilities were analyzed with the MTT assay 24 hours after minocycline (0 to 10 μ*M*) treatment. Minocycline did not affect hBM-MSC viability until 10 μ*M*, whereas high concentrations (8 to 10 μ*M*) decreased astrocyte viability. Points, mean; bars, SEM. **(B)** FACS analysis of the effect of minocycline on hBM-MSC phenotype. Wild-type (black lines) and minocycline-treated hBM-MSCs (red lines) were labeled with antibodies for MSC phenotypic surface markers. Minocycline did not change surface-marker expression; hBM-MSCs expressed CD90, CD44, and CD73 and lacked CD34, CD45, and HLA-DR. **(C)** The effect of minocycline on the differentiation potential of hBM-MSCs. Minocycline did not affect the differentiation capability of hBM-MSCs to adipogenic or osteogenic lineages, as stained by Oil Red O and Alizarin Red S, respectively. The results are representative of three independent experiments.

To examine whether minocycline could affect the viability of hBM-MSCs and astrocytes, these cells were grown in media containing various concentrations of minocycline. The viability of hBM-MSCs was not affected until 10 μ*M*, and 8 to 10 μ*M* decreased astrocyte viability (Figure 1A). The evident toxicity to astrocytes, which is a representative cell type of the CNS, prompted the use of a lower dose of minocycline for the following *in vivo* combination experiments. In addition, to investigate the characteristic features of minocycline-treated hBM-MSCs, we evaluated the surface phenotypes of hBM-MSCs with FACS. Similar to wild-type hBM-MSCs, minocycline-treated hBM-MSCs were strongly positive for CD90, CD44, and CD73, and negative for CD34, CD45, and HLA-DR (Figure 1B). The FACS analysis did not reveal a significant difference between wild-type hBM-MSCs and minocycline-treated hBM-MSCs in the number of surface markers (Figure 1B). The effect of minocycline on hBM-MSCs differentiation potential was assessed by culturing the cells in induction medium with or without minocycline (10 μ*M*) for 3 to 4 weeks. Minocycline did not affect the differentiation ability to adipogenic or osteogenic lineages (Figure 1C). Furthermore, minocycline-treated hBM-MSCs migrated and engrafted into the spinal cords of EAE mice after intravenous administration (see Additional file 1: Figure S1). Taken together, these results suggest that the viability and characterization of hBM-MSCs are not affected by minocycline.

### Combined treatment improves the clinical score of EAE mice

**Figure 2:**
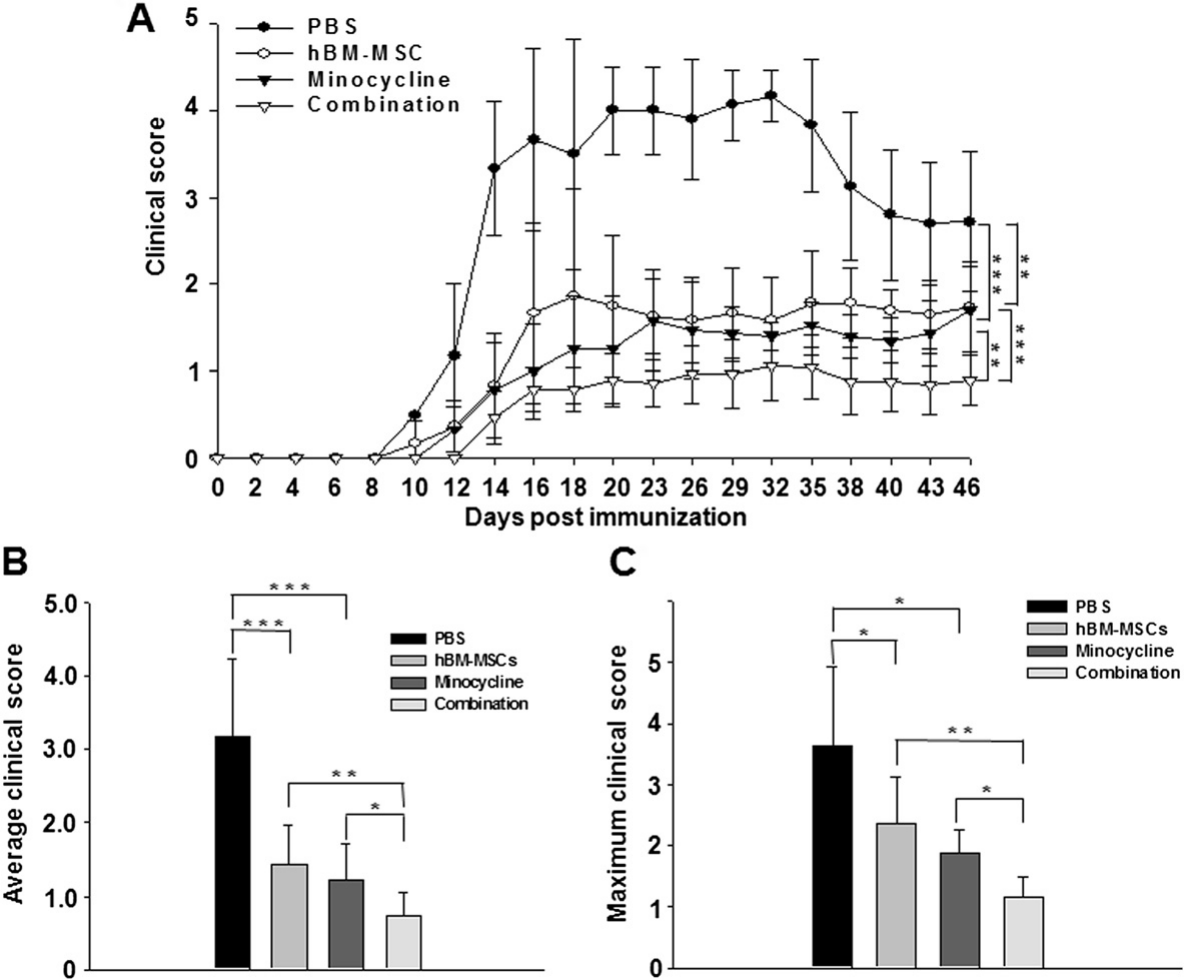
**The combination of hBM-MSCs and minocycline attenuates clinical EAE severity in mice.** EAE was induced in female C57 BL/6 mice by immunization with the MOG 35- to 55-amino acid peptide. **(A)** Mean daily clinical scores for the different EAE treatments (*n* = 10 per group) revealed that PBS-treated mice developed EAE with an average maximum severity over grade 4. Treatment with hBM-MSCs or minocycline alone decreased disease severity, with an average maximum severity of grades 1 to 2. Combination treatment markedly attenuated disease severity, with an average maximum severity of grade 1. Points, mean; bars, SEM. **(B)** The average clinical score of the four groups was assessed from day 1 until day 50 after immunization. Compared with PBS treatment, hBM-MSCs or minocycline treatment alone significantly decreased the average clinical scores (*P* < 0.05). Compared with hBM-MSCs or minocycline treatment alone, combination treatment significantly decreased the average clinical scores (*P* < 0.05). **(C)** The maximum clinical score for each mouse over the course of the entire experiment was recorded, and the trend of the maximum clinical scores in the four groups was the same as the average clinical score (*P* < 0.05). Columns, mean; bars, SEM. *P < 0.05, **P < 0.01, ***P < 0.001, one-way ANOVA with *post hoc* Bonferroni corrections. The results are representative of three independent experiments.

To evaluate the effect of combination treatment with hBM-MSCs and minocycline on disease progression in EAE mice, we injected PBS, hBM-MSCs, minocycline, or a combination of hBM-MSCs and minocycline into mice 7 days after immunization (*n* = 10/group). Neurologic function was tested daily until 50 days after immunization. PBS-treated mice developed EAE with clinical symptoms onset 10 days after immunization. Compared with PBS treatment, treatment with hBM-MSCs or minocycline postponed the onset of the clinical symptoms. hBM-MSCs-treated mice developed EAE with clinical symptoms an average of 12.0 ± 1.80 days after immunization (*P* = 0.0035, PBS versus hBM-MSCs treatment). Minocycline-treated mice developed EAE with clinical symptoms an average of 12.67 ± 1.0 days after immunization (*P* < 0.001, PBS versus minocycline treatment). The combination treatment significantly postponed clinical symptom onset compared with either single treatment; the mice developed EAE an average of 14.5 ± 1.0 days after immunization (*P* < 0.001, hBM-MSCs versus combination treatment; *P* = 0.007, minocycline versus combination treatment) (Figure 2A). Compared with PBS treatment, treatment with hBM-MSCs or minocycline significantly decreased the average clinical scores (average clinical score in the PBS treatment, 3.166 ± 1.075; hBM-MSCs treatment, 1.433 ± 0.534; minocycline treatment, 1.223 ± 0.4936; *P* < 0.001, PBS versus hBM-MSCs treatment; *P* < 0.001, PBS versus minocycline treatment) and the maximum clinical scores (PBS treatment, 3.640 ± 1.284; hBM-MSCs treatment, 2.333 ± 0.6831; minocycline treatment, 2.033 ± 0.2582; *P* = 0.028; PBS versus hBM-MSCs treatment; *P* = 0.014, PBS versus minocycline treatment). The decreases in the combination-treatment group were much more significant compared with either single treatment throughout the whole disease course (average clinical scores of the combination-treatment group, 0.7375 ± 0.3235, *P* = 0.005; hBM-MSCs versus combination treatment, *P* = 0.031; minocycline versus combination treatment; maximal clinical score of the combination-treatment group: 1.1625 ± 0.3292, *P* = 0.009 hBM-MSCs versus combination treatment; *P* = 0.024, minocycline versus combination treatment) (Figure 2B, C). These results suggest that combination treatment with hBM-MSCs and minocycline ameliorates clinical severity and improves neurologic functional recovery of EAE mice.

### Combined treatment reduces the number of inflammatory cells in EAE mouse spinal cord

**Figure 3:**
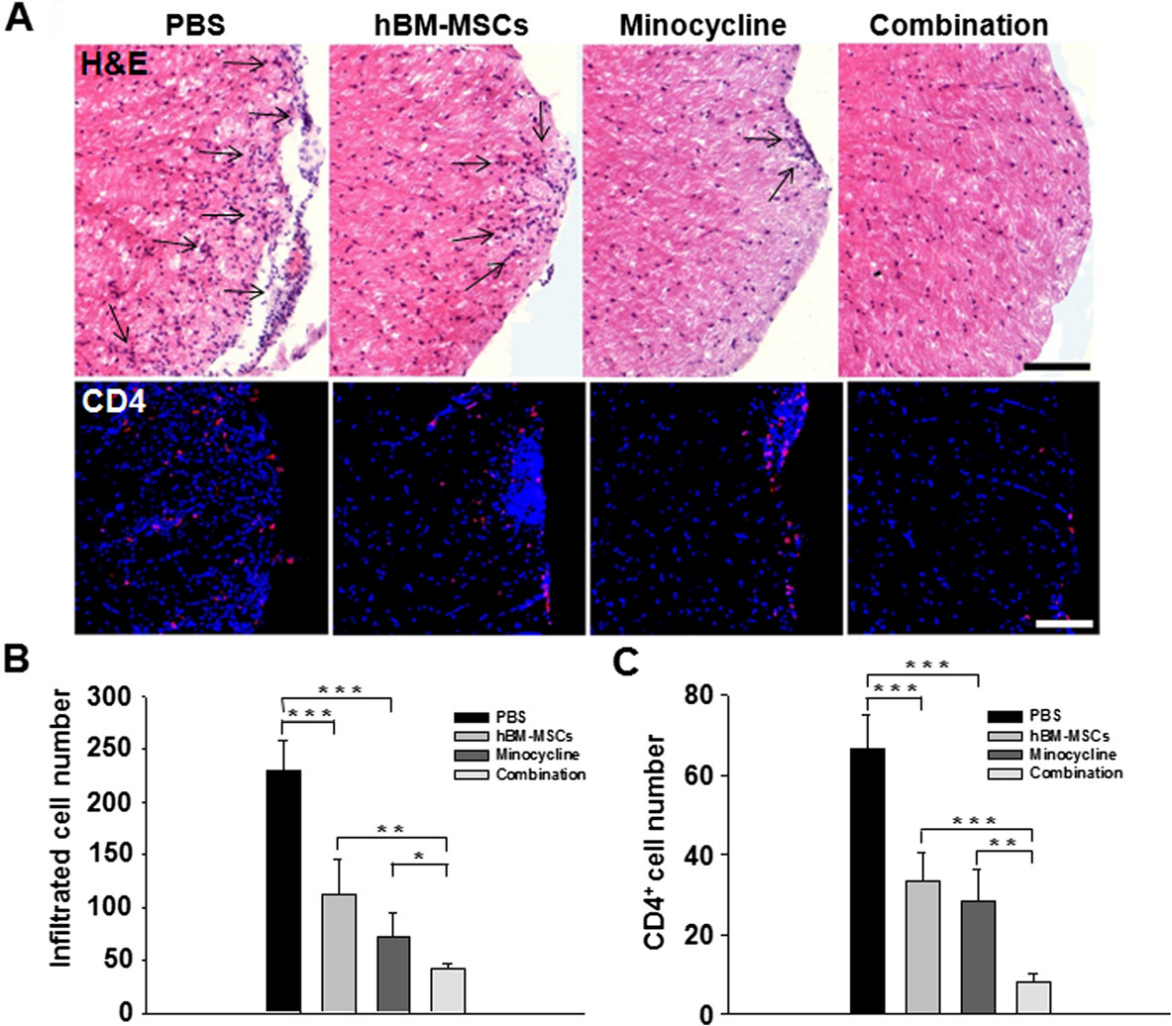
**The combination of hBM-MSCs and minocycline decreases inflammation in EAE mouse spinal cord. (A)** H&E (upper panel) and CD4 staining (bottom panel) were performed to detect mononuclear and T-cell infiltration in the lumbar spinal cords of EAE mice. The arrows indicate areas with infiltrated cells. Scale bar, 200 μm. **(B)** Statistical analysis showing that compared with PBS treatment, hBM-MSCs or minocycline treatment alone significantly decreased the average clinical scores (*P* < 0.05); compared with hBM-MSCs or minocycline treatment alone, combination treatment significantly decreased the numbers of infiltrating cells by 20% to 30% (*P* < 0.05). **(C)** Compared with hBM-MSCs or minocycline treatment alone, combination treatment significantly decreased the numbers of CD4^+^ cells by 30% to 40% (*P* < 0.05). Columns, mean; bars, SEM. *P < 0.05, **P < 0.01, ***P < 0.001, one-way ANOVA with *post hoc* Bonferroni corrections. The results are representative of three independent experiments.

The improvement in clinical scores after combined treatment may reflect decreased inflammatory cell infiltration into the CNS. To identify the effect of combination treatment on inflammatory-cell influx, sections of lumbar spinal cords from EAE mice were labeled with H&E and anti-CD4 to detect mononuclear cell and T-cell infiltration, respectively. Infiltrated cells were increased in spinal cord white matter in the PBS-treatment group. However, these cells were reduced in number after combined treatment compared with both single treatments (Figure 3A). Stereologic analysis demonstrated that hBM-MSCs or minocycline treatment alone decreased the numbers of infiltrating cells compared with the PBS treatment (*P* < 0.001, PBS versus hBM-MSCs treatment; *P* < 0.001, PBS versus minocycline treatment), and a further significant decrease occurred in combination treatment mice compared with the hBM-MSCs or minocycline treatment alone (*P* = 0.002, hBM-MSCs versus combination treatment; *P* = 0.020, minocycline versus combination treatment) (Figure 3B). Furthermore, a significant decrease in CD4^+^ T-cell infiltration in the spinal cord of combination-treatment mice was evident compared with single treatment with hBM-MSCs or minocycline (*P* < 0.001, hBM-MSCs versus combination treatment: *P* = 0.003, minocycline versus combination treatment) (Figure 3C).

### Combined treatment reduces demyelination in the EAE mouse spinal cord

**Figure 4:**
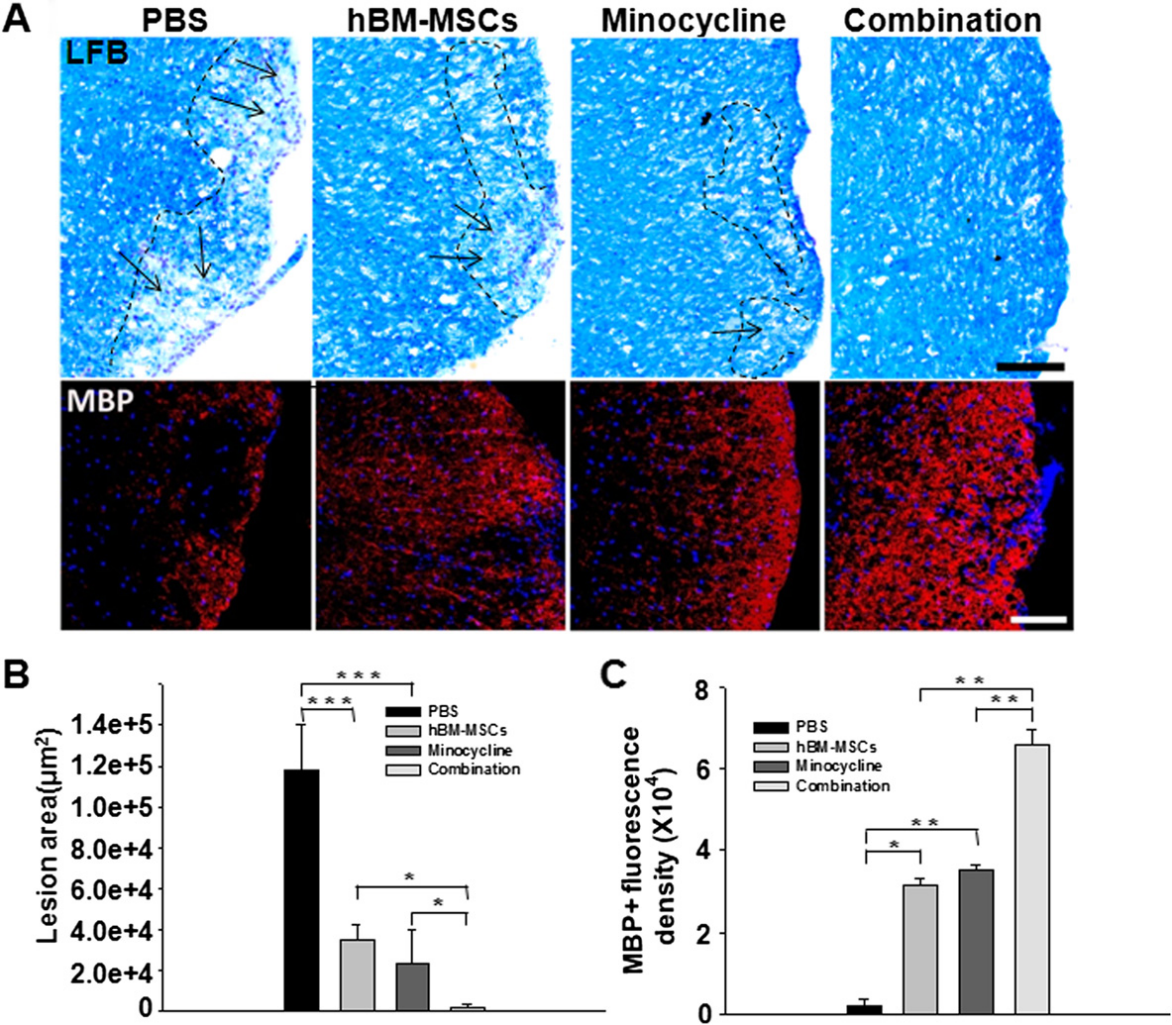
**The combination of hBM-MSCs and minocycline decreases demyelination in the EAE mouse spinal cord. (A)** LFB- (upper panel) and MBP-stained (bottom panel) spinal cord sections were assessed to detect the extent of demyelination in EAE mice. The arrows indicate demyelinated areas. Scale bar, 200 μm. **(B)** Statistical analysis demonstrated that combination treatment significantly reduced demyelination compared with hBM-MSCs or minocycline treatment alone (*P* < 0.05). **(C)** Combination treatment also significantly preserved the number of MBP-positive cells compared with hBM-MSCs or minocycline treatment alone (*P* < 0.001). Columns, mean; bars, SEM. *P < 0.05, **P < 0.01, ***P < 0.001, one-way ANOVA with *post hoc* Bonferroni corrections. The results are representative of three independent experiments.

To determine whether combination treatment reduced tissue damage in EAE mice, sections of lumbar spinal cords were labeled with LFB and anti-MBP. Demyelination was observed in lumbar spinal cord white matter and was reduced to a greater extent by combined treatment compared with either single treatment (Figure 4A). Stereologic analysis of the extent of demyelination revealed a significant reduction in mice treated solely with hBM-MSCs or minocycline compared with the PBS-treatment group (*P* < 0.001, PBS versus hBM-MSCs treatment; *P* < 0.001, PBS versus minocycline treatment). Moreover, a significant decrease was found in the combination-treatment group compared with both single treatments (*P* < 0.001, PBS versus combination treatment; *P* = 0.017, hBM-MSCs versus combination treatment; *P* = 0.038, minocycline versus combination treatment) (Figure 4B). Furthermore, immunofluorescence staining revealed significant preservation of MBP expression in the spinal cords of the hBM-MSCs or minocycline treatment groups compared with mice that received PBS (*P* = 0.048, PBS versus hBM-MSCs treatment; *P* = 0.007, PBS versus minocycline treatment. The intensity of labeling for MBP-positive cells significantly increased in the combination-treatment group compared with hBM-MSCs or minocycline treatment alone (*P* = 0.005, hBM-MSCs versus combination treatment; *P* = 0.003, minocycline versus combination treatment) (Figure 4C). Thus, these results suggest that combination treatment with hBM-MSCs and minocycline reduced tissue damage and alleviated the clinical symptoms of EAE.

### Combined treatment reduces neuroinflammation and neurodegeneration in EAE mouse spinal cord

**Figure 5:**
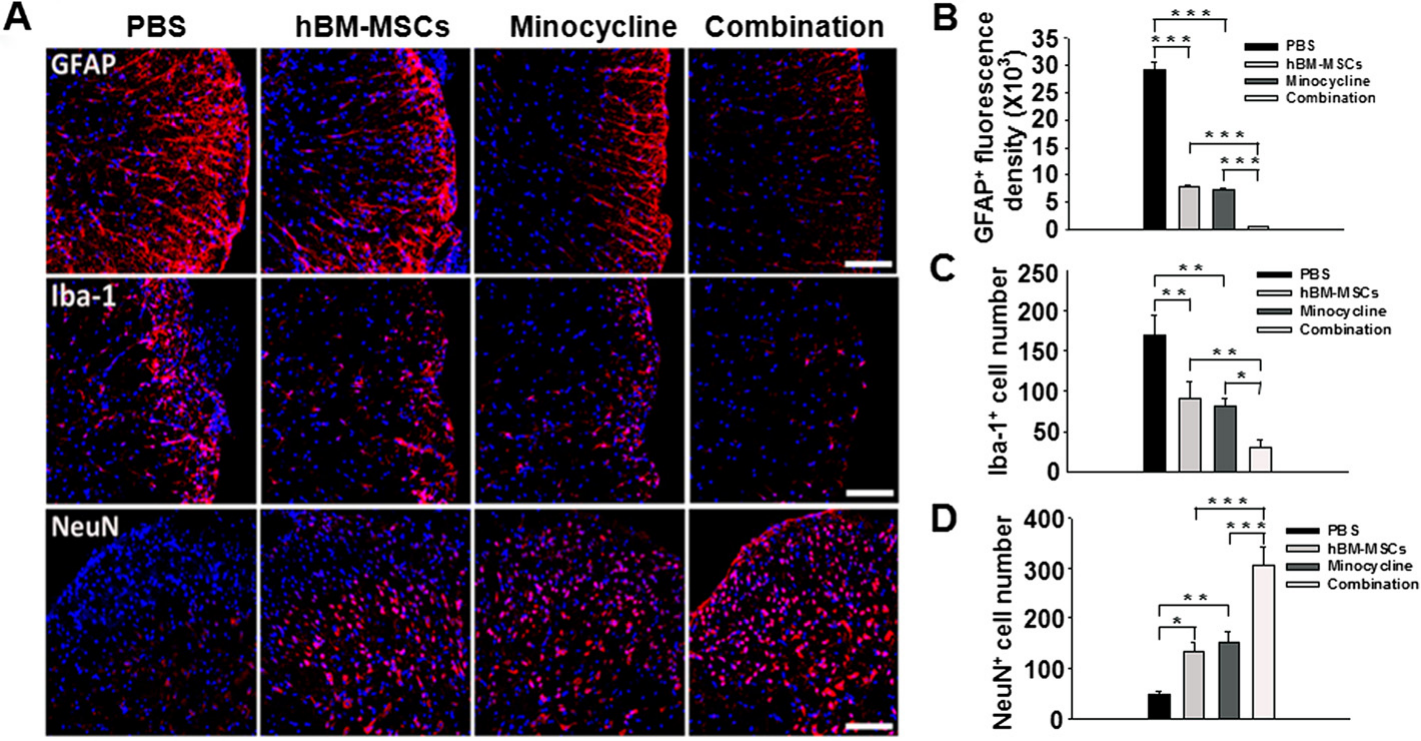
**Combined treatment reduces glial reactivity and protects neurons in the EAE mouse spinal cord. (A)** Sections are labeled for GFAP (upper panel), Iba-1 (middle panel), and NeuN (bottom panel) immunoreactivity to detect astrocytes, microglia, and neurons, respectively. Intense GFAP immunoreactivity was present in PBS-treated EAE mice and was reduced in EAE mice treated with hBM-MSCs or minocycline alone, but fewer astroglial cells were activated in the combination-treatment group. Iba-1, which identifies activated microglia, displayed the same immunoreactivity patterns as GFAP in the four EAE groups. The opposite pattern was observed for the neuronal marker, NeuN, in all groups. Scale bar, 200 μm. **(B)** Stereologic analyses of GFAP fluorescence intensity and **(C)** the number of Iba-1-positive cells revealed significant reductions in astrocytes and microglial activation in combination-treatment mice compared with those treated with hBM-MSCs or minocycline alone (*P* < 0.05). **(D)** Stereologic analysis also revealed a significant increase in the number of NeuN-positive cells in spinal cord sections of EAE mice treated with both hBM-MSCs and minocycline compared with hBM-MSCs or minocycline treatment alone (*P* < 0.01). Columns, mean; bars, SEM. *P < 0.05, **P < 0.01, ***P < 0.001, one-way ANOVA with *post hoc* Bonferroni corrections. The results are representative of three independent experiments.

Neuroinflammation and neurodegeneration were evaluated by analyzing GFAP-, Iba-1-, and NeuN-positive cells. In PBS-treated EAE mice, intense glial activation with increases in the number of cells intensely stained for GFAP and Iba-1 in the lesion area was evident. In EAE mice treated with hBM-MSCs or minocycline alone, we observed a decreased number of astrocytes and microglia cells compared with PBS-treated EAE mice. Only a small number of GFAP- and Iba-1-positive astrocytes and microglia cells, respectively, were observed in the combination-treatment mice (Figure 5A). Stereologic analyses of GFAP- and Iba-1-positive cells demonstrated significant reductions in the fluorescence intensity of labeling for activated astrocytes and the number of activated microglia cells after treatment with hBM-MSCs or minocycline alone, compared with the PBS treatment (GFAP: *P* < 0.001, PBS versus hBM-MSCs treatment; *P* < 0.001, PBS versus minocycline treatment; Iba-1: *P* = 0.002, PBS versus hBM-MSCs treatment; *P* = 0.001, PBS versus minocycline treatment). A significant reduction was noted in the combination-treatment group compared with either single treatment (GFAP: *P* < 0.001, hBM-MSCs versus combination treatment; *P* < 0.001, minocycline versus combination treatment; Iba-1: *P* = 0.009, hBM-MSCs versus combination treatment; *P* = 0.024, minocycline versus combination treatment) (Figure 5B, C).

Next, we evaluated the neuroprotection conferred by each treatment by counting the number of neurons in the gray matter of lumbar spinal cord sections. The number of NeuN-positive neurons increased in the gray matter of EAE mice treated with hBM-MSCs or minocycline alone compared with the PBS treatment, whereas the combination treatment induced a marked increase in NeuN immunoreactivity. Stereologic analysis verified a significant increase in the number of NeuN-positive cells in spinal cord sections of EAE mice treated with hBM-MSCs or minocycline alone compared with the PBS treatment (*P* = 0.016, PBS versus hBM-MSCs treatment; *P* = 0.004, PBS versus minocycline treatment). However, compared with the treatment with hBM-MSCs or minocycline alone, a significant increase was noted in the combination-treatment group (*P* < 0.001, hBM-MSCs versus combination treatment; *P* < 0.001, minocycline versus combination treatment) (Figure 5D). Taken together, these results suggest that combination treatment with hBM-MSCs and minocycline alleviates neurodegeneration and reduces neuroinflammation in the CNS, mitigates the clinical symptoms of EAE.

### Combined treatment promotes a shift from Th1 to Th2 cytokine balance in EAE mice

**Figure 6:**
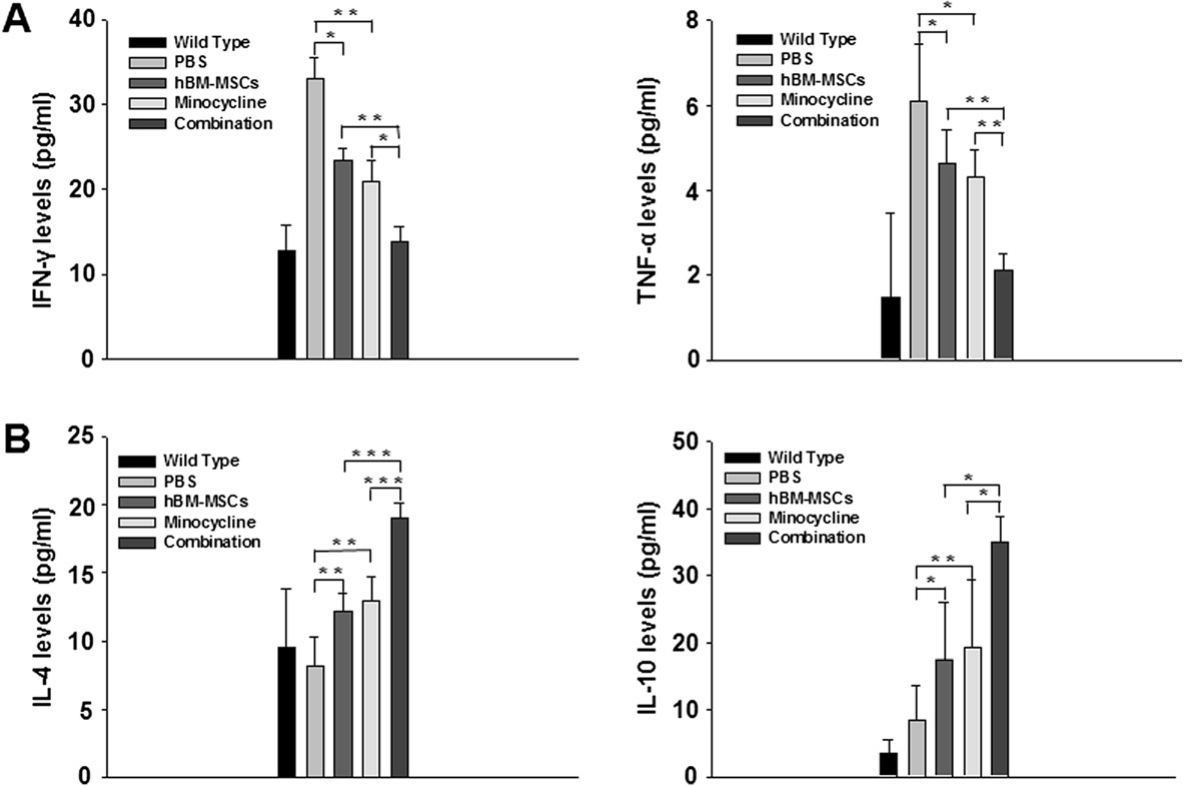
**Combination treatment promotes a shift from the Th1 to Th2 cytokine balance in EAE mice.** Sera were isolated from the five groups of EAE mice 40 days after immunization. Cytokine protein-expression levels in the serum were quantified with ELISA. **(A)** Stereologic analysis demonstrated a significant decrease of IFN-γ/TNF-α Th1 cytokines in the hBM-MSCs or minocycline-treatment groups compared with PBS treatment, and a similar expression pattern was evident in the combination-treatment group compared with hBM-MSCs or minocycline treatment-alone groups. **(B)** Significant increases were present in IL-4 and IL-10 protein-expression levels in the hBM-MSCs or minocycline-treatment groups compared with PBS treatment, and the same pattern was evident in the combination-treatment mice compared with hBM-MSCs or minocycline treatment alone. Columns, mean; bars, SEM. *P < 0.05, **P < 0.01, ***P < 0.001, one-way ANOVA with *post hoc* Bonferroni corrections. The results are representative of three independent experiments.

To determine whether the combination treatment would modulate inflammatory cytokine expression, we evaluated the systemic expression of IFN-γ/TNF-α and IL-4/IL-10 cytokines in EAE mice sera with ELISA. We observed a significant decrease of IFN-γ/TNF-α Th1 cytokines and an increase of IL-4/IL-10 Th2 cytokines expression in the hBM-MSCs or minocycline treatment groups compared with the PBS-treatment group (IFN-γ: *P* = 0.010, PBS versus hBM-MSCs treatment; *P* = 0.005, PBS versus minocycline treatment; TNF-α: *P* = 0.042, PBS versus hBM-MSCs treatment; *P* = 0.009, PBS versus minocycline treatment; IL-4: *P* = 0.004, PBS versus hBM-MSCs treatment; *P* = 0.001, PBS versus minocycline treatment; IL-10: *P* = 0.022, PBS versus hBM-MSCs treatment; *P* = 0.008, PBS versus minocycline treatment) (Figure 6). In addition, we detected a significant decrease of IFN-γ/TNF-α Th1 cytokines and an increase of IL-4/IL-10 Th2 cytokines in the combination treatment group compared with both single-treatment groups (IFN-γ: *P* = 0.006, hBM-MSCs versus combination treatment; *P* = 0.048, minocycline versus combination treatment; TNF-α: *P* = 0.006, hBM-MSCs versus combination treatment; *P* = 0.009, minocycline versus combination treatment; IL-4: *P* < 0.001, hBM-MSCs versus combination treatment; *P* < 0.001, minocycline versus combination treatment; IL-10: *P* = 0.028, hBM-MSCs versus combination treatment; *P* = 0.043, minocycline versus combination treatment) (Figure 6).

Furthermore, because the initial immune reactions take place in the periphery before immune cell migration into the CNS, we examined the effect of all treatments on the systemic immune microenvironment in an *in vitro* system. IFN-γ and IL-4 production were assessed in anti-MOG35-55-stimulated splenocytes isolated from all EAE groups. The analysis revealed a significant decrease of IFN-γ and a significant increase of IL-4 production in the hBM-MSCs or minocycline treatment groups compared with the PBS-treatment group (IFN-γ: *P* = 0.006, PBS versus hBM-MSCs treatment; *P* < 0.001, PBS versus minocycline treatment; IL-4: *P* = 0.042, PBS versus. hBM-MSCs treatment; *P* = 0.035, PBS versus. minocycline treatment). The same pattern was also detected in the combination treatment, but the effect was significantly greater than observed in the single treatment groups (IFN-γ: *P* = 0.025, hBM-MSCs versus combination treatment; *P* = 0.037, minocycline versus combination treatment; IL-4: *P* = 0.039, hBM-MSCs versus combination treatment; *P* = 0.046, minocycline versus combination treatment) (see Additional file 2: Figure S2). These results suggest that the combination treatment of hBM-MSCs and minocycline systemically affect the effector phase of EAE. Collectively, it appears that a significant shift occurs from the Th1 to Th2 cytokine balance in the combination-treatment EAE mice.

### Combined treatment reduces apoptotic cell death in EAE mouse spinal cord

**Figure 7:**
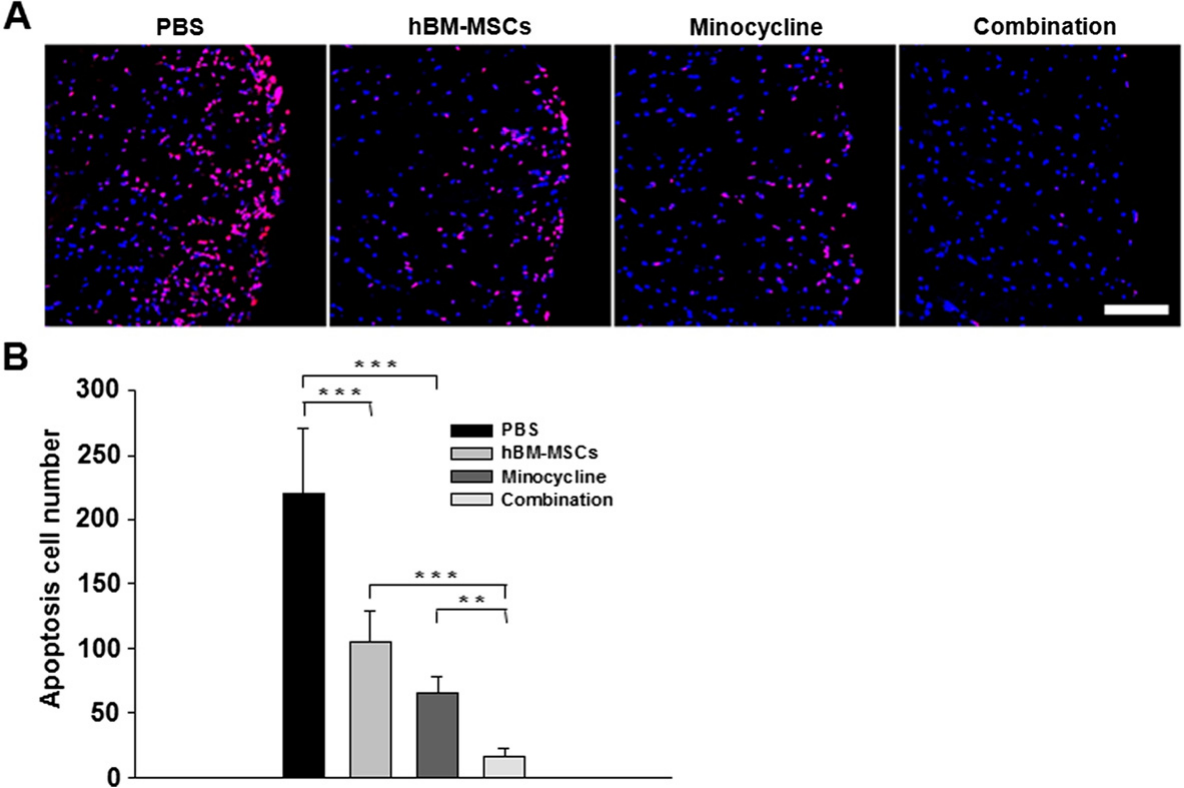
**hBM-MSCs and minocycline in combination reduce apoptosis in EAE mouse spinal cord. (A)** Apoptotic cell death was examined with TUNEL staining. TUNEL-positive cells are red, and counterstaining for DAPI is blue. Scale bar, 200 μm. **(B)** TUNEL-positive cells were also quantified with MetaMorph image analysis. A statistically significant reduction in the number of apoptotic cells in the hBM-MSCs or minocycline treatment-alone group compared with the PBS-treated group (*P* < 0.001), and the combination treatment showed a more significant decrease in apoptosis compared to hBM-MSCs or minocycline treatment alone (*P* < 0.001). Columns, mean; bars, SEM. **P < 0.01, ***P < 0.001, one-way ANOVA with *post hoc* Bonferroni corrections. The results are representative of three independent experiments.

To investigate whether the combination treatment could protect injured spinal cord cells from apoptosis, apoptotic cell death was examined with TUNEL staining. The number of apoptotic cells was reduced in the single-treatment groups compared with the PBS-treated group (*P* < 0.001, PBS versus hBM-MSCs treatment; *P* < 0.001, PBS versus minocycline treatment), but the combination treatment group exhibited a more significant decrease in the number of apoptotic cells compared with the groups treated with hBM-MSCs or minocycline alone (*P* < 0.001, hBM-MSCs versus combination treatment; *P* = 0.002, minocycline versus combination treatment) (Figure 7). Thus, during EAE, apoptotic cell death in the lesion sites was reduced by combination treatment. In addition, we confirmed that most apoptotic cells were double labeled with NeuN, the neuronal marker, whereas only a few were positive for GFAP, Iba-1, or CD4 (see Additional file 3: Figure S3). These results suggest that combination treatment could decrease neuronal death in EAE mouse spinal cord.

## Discussion

A therapeutic approach to improve MS treatment is to identify an effective combination of new medications or existing therapies that affect different aspects of the disease process and mitigate the adverse events by using lower doses of individual drugs as a combination therapy [22]. One issue to consider is that such a combination could synergistically produce adverse effects or events. Minocycline has multiple immunomodulatory and neuroprotective activities [23], but it can also cause side effects, such as systemic lupus erythematosus and serum sickness [9,10] and is toxic to the nervous system [11,12]. We found that high doses of minocycline decreased astrocyte viability. We also tested the effects of minocycline on hBM-MSCs and found that it did not affect their viability or characteristics, even at a high dose. As hBM-MSCs and minocycline meet the combined-therapy criteria, it is reasonable to test their combination-therapy efficacy in MS.

Presently, although hBM-MSCs or a low-dose minocycline treatment alone had significant effects on EAE mice compared with PBS treatment, the combination treatment further protected EAE mice from disease progression along with significant attenuation of disease severity, reductions in inflammatory infiltration, demyelination, neurodegeneration, and enhancement of the immunomodulatory function. Many possible mechanisms exist for the effects of combination treatment: the first beneficial effect of the combination treatment might involve modulation of the expression/production of IFN-γ/TNF-α and IL-4/IL-10 in the serum and splenocyte cultures of EAE mice in this study.

Cytokines play an important role in MS pathogenesis, as well as in EAE [24]. The balance between Th1 and Th2 in the CNS may be the key determinant in the development of EAE, which is a Th1-mediated disease [25,26]. In contrast, Th2 cytokines have been associated with remission and recovery [27]. In organ-specific autoimmunity, the cytokine balance is pivotal in the determination of resistance or susceptibility [28,29]. EAE susceptibility is thought to correlate with the expression of IFN-γ and TNF-α, which are the primary proinflammatory Th1 cytokines, whereas Th2 cytokines, such as IL-4 and IL-10, are antiinflammatory cytokines important for preventing or ameliorating disease [30]. TNF-α/IFN-γ and IL-4/IL-10 are considered the hallmark cytokines that direct Th1 and Th2 development and play an important role in both MS and EAE pathogenesis. Our finding that the combination treatment promoted a shift from the Th1 to Th2 cytokine balance in EAE mice is in accordance with previous observations that minocycline modulates immune differentiation from a Th1 toward a Th2 phenotype; therefore reducing T-cell infiltration into the spinal cord in MS and EAE [2,5,31]. Furthermore, hBM-MSCs are able to suppress T-lymphocyte activation and proliferation, induce Th2-polarized immune response, and promote endogenous repair, thus exerting immunomodulatory effects both *in vitro* and *in vivo*[14-17]. Our observations that the combination treatment of hBM-MSCs and minocycline exceeded the effects of either treatment alone indicate effective synergistic function.

The second possible mechanism is inhibition of inflammation and glial activation. Inflammation is considered a cause of tissue damage during relapsing-remitting MS and EAE. Therefore, antiinflammation continues to be the primary therapeutic objective during early MS [32]. Glial activation is thought to play a crucial role in tissue destruction through the production of proinflammatory cytokines and massive proliferation that may overwhelm the surrounding cells. In our study, hBM-MSCs or minocycline alone, as well as combination treatment, significantly decreased mononuclear and T-cells infiltration, and microglial and astrocyte activation. The data presented here agree with the previous observation that inhibition of glial cell activation ameliorates EAE severity [33] and that minocycline is neuroprotective by inhibiting microglial activation [8,34,35]. Transplanted hBM-MSCs provide a feasible and practical means of neuroprotection by decreasing microglial and astrocyte activation; they also reduce apoptosis by secreting neurotrophic factors, remyelinating focal demyelination lesions in the spinal cord, and improving functional outcome after spinal cord injury [18,36-40]. These may also be important factors to enhance the therapy effect of this combination treatment in EAE mice.

In addition, a recent study reported that minocycline induces neuroprotection not due to its antiinflammatory action, but directly through the induction of antiapoptotic intracellular signaling pathways [4]. These observations are consistent with the present observation that combined treatment with hBM-MSCs and minocycline significantly decreased apoptosis in the lesion sites of EAE mice.

## Conclusions

The combination of hBM-MSCs and minocycline exerts effective therapy effects by promoting a shift from Th1 to Th2 cytokine balance, decreasing inflammatory cell influx, suppressing demyelination, reducing apoptosis, enhancing neuroprotection, and preventing disease progression in EAE mice. Our results indicate that this therapeutic strategy is a promising approach for the treatment of MS.

## Abbreviations

EAE: Experimental autoimmune encephalomyelitis; GFAP: Glial fibrillary acidic protein; hBM-MSCs: Human bone marrow mesenchymal stem cells; Iba1: Ionized calcium-binding adaptor molecule 1; IFN-γ: Interferon gamma; IL-4: Interleukin-4; IL-10: Interleukin-10; MBP: Myelin basic protein; MS: Multiple sclerosis; NeuN: Neuronal nuclear antigen; TdT: Terminal deoxynucleotidyl transferase; TNF-α: Tumor necrosis factor alpha.

## Competing interests

The authors declare that they have no competing interests.

## Authors’ contributions

YH participated in the design of the study, performed most of the experiments, collected data, and analyzed statistical data analysis. GYP participated in collecting the animal samples. SMK prepared the hBM-MSCs and tested the cell viability *in vitro*. CHJ carried out the threshold image analysis of the spinal cord. SSJ and CHR contributed to the conception and design of the study, interpretation of data, and editing of the manuscript. All authors read and approved the final manuscript.

## Supplementary Material

Additional file 1: Figure S1 Identification of minocycline-treated hBM-MSCs in the spinal cord of EAE mice. hBM-MSCs infected with Ad-GFP (50 MOI) were treated with minocycline (10 μ*M*). Five days after minocycline-treated hBM-MSCs (1.5 × 10^6^) administration, hBM-MSCs were visualized by using a Zeiss LSM 700 confocal microscope. **(A)** The GFP-transduced hBM-MSCs (green) distributed in the lesion area; many of them were closely associated with blood vessels. **(a)** The high magnification of the boxed area in **(A)**. Nuclei were counterstained with DAPI (blue). The arrows indicate GFP-positive cells. Scale bar = 1 mm in **(A)**, Scale bar = 200 μm in **(a)**. Click here for file

Additional file 2: Figure S2 Combined treatment reduces frequency of Th1 but increases frequency of Th2 in EAE mice. Splenocytes (5 × 10^5^/well) isolated from EAE mice at day 40 after immunization were stimulated with MOG35-55. The number of MOG-specific IFN-γ (A)/IL-4 (B) producing splenocytes was determined with enzyme-linked immunospot (ELISPOT) assay. Combination treatment significantly decreased frequencies of Th1, but increased frequencies of myelin peptide-specific Th2 compared with hBM-MSCs or minocycline treatment alone (*P* < 0.05). The wells shown are representatives of triplicates. Columns, mean; bars, SEM. *P < 0.05, **P < 0.01, ***P < 0.001, one-way ANOVA with *post hoc* Bonferroni corrections. The results are representative of three independent experiments. Click here for file

Additional file 3: Figure S3 Characterization of the apoptotic cells in the lumbar spinal cords of the EAE mice. **(A)** Sections were double labeling of TUNEL (red) with NeuN (green) in the white matter of the lumbar spinal cords. **(a)** High magnification of the boxed area in **(A)**; most apoptotic cells were positive for NeuN (arrows in a). **(B)** Double labeling of TUNEL (red) with GFAP (green) in the white matter of the lumbar spinal cords. **(b)** High magnification of the boxed area in **(B)**; a few apoptotic cells were positive for GFAP (arrows in b). **(C)** Double labeling of TUNEL (red) with Iba-1 (green) in the white matter of the lumbar spinal cords. **(c)** High magnification of the boxed area in **(C)**; a few apoptotic cells were positive for Iba-1 (arrows in c). **(D)** Double labeling of TUNEL (red) with CD4 (green) in the white matter of the lumbar spinal cords. **(d)** High magnification of the boxed area in **(D)**; a few apoptotic cells were positive for CD4 (arrows in d). Nuclei were counterstained with DAPI (blue). The arrows indicate positive cells. Scale bars = 200 μm in (A through D), Scale bars = 100 μm in (a through d). Click here for file
