# Supplementary figures and images for: Effective combination of human bone marrow mesenchymal stem cells and minocycline in experimental autoimmune encephalomyelitis mice
Source: Stem Cell Res Ther. 2013 Jul 5;4(4):77. doi: 10.1186/scrt228 (PMC3854709; doi:10.1186/scrt228)

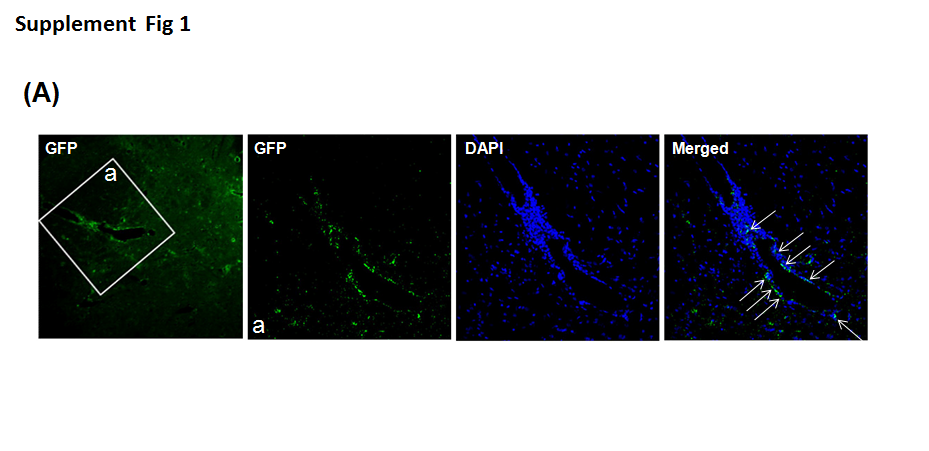

Supplement: Additional file 1: Figure S1 — Identification of minocycline-treated hBM-MSCs in the spinal cord of EAE mice. hBM-MSCs infected with Ad-GFP (50 MOI) were treated with minocycline (10 μM). Five days after minocycline-treated hBM-MSCs (1.5 × 10^6^) administration, hBM-MSCs were visualized by using a Zeiss LSM 700 confocal microscope. (A) The GFP-transduced hBM-MSCs (green) distributed in the lesion area; many of them were closely associated with blood vessels. (a) The high magnification of the boxed area in (A). Nuclei were counterstained with DAPI (blue). The arrows indicate GFP-positive cells. Scale bar = 1 mm in (A), Scale bar = 200 μm in (a). [file scrt228-S1.tiff]

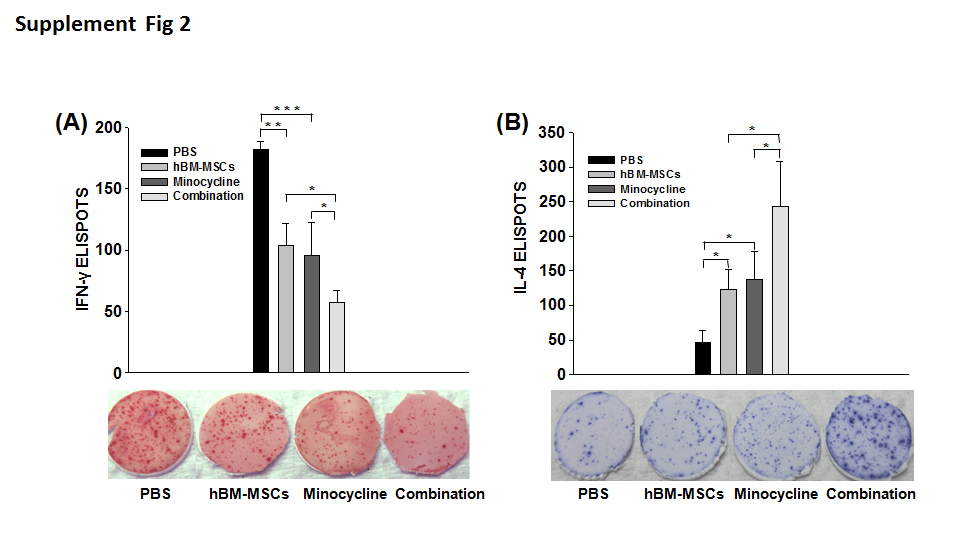

Supplement: Additional file 2: Figure S2 — Combined treatment reduces frequency of Th1 but increases frequency of Th2 in EAE mice. Splenocytes (5 × 10^5^/well) isolated from EAE mice at day 40 after immunization were stimulated with MOG35-55. The number of MOG-specific IFN-γ (A)/IL-4 (B) producing splenocytes was determined with enzyme-linked immunospot (ELISPOT) assay. Combination treatment significantly decreased frequencies of Th1, but increased frequencies of myelin peptide-specific Th2 compared with hBM-MSCs or minocycline treatment alone (P < 0.05). The wells shown are representatives of triplicates. Columns, mean; bars, SEM. *P < 0.05, **P < 0.01, ***P < 0.001, one-way ANOVA with post hoc Bonferroni corrections. The results are representative of three independent experiments. [file scrt228-S2.tiff]

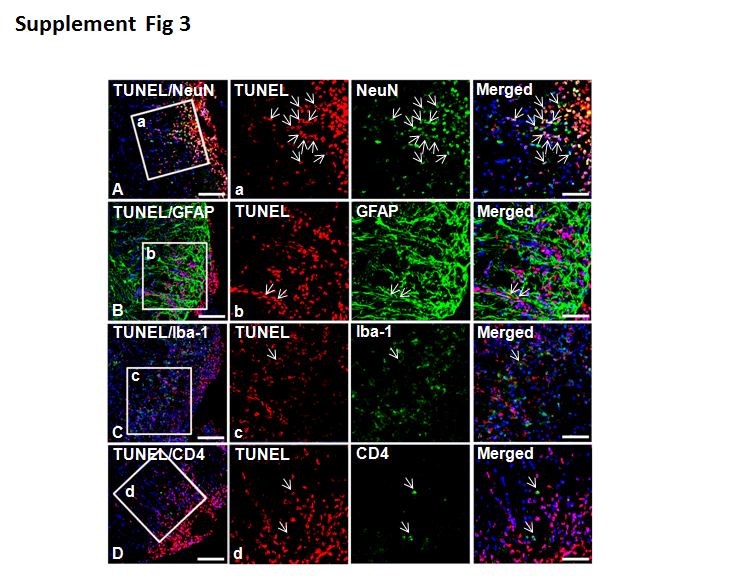

Supplement: Additional file 3: Figure S3 — Characterization of the apoptotic cells in the lumbar spinal cords of the EAE mice. (A) Sections were double labeling of TUNEL (red) with NeuN (green) in the white matter of the lumbar spinal cords. (a) High magnification of the boxed area in (A); most apoptotic cells were positive for NeuN (arrows in a). (B) Double labeling of TUNEL (red) with GFAP (green) in the white matter of the lumbar spinal cords. (b) High magnification of the boxed area in (B); a few apoptotic cells were positive for GFAP (arrows in b). (C) Double labeling of TUNEL (red) with Iba-1 (green) in the white matter of the lumbar spinal cords. (c) High magnification of the boxed area in (C); a few apoptotic cells were positive for Iba-1 (arrows in c). (D) Double labeling of TUNEL (red) with CD4 (green) in the white matter of the lumbar spinal cords. (d) High magnification of the boxed area in (D); a few apoptotic cells were positive for CD4 (arrows in d). Nuclei were counterstained with DAPI (blue). The arrows indicate positive cells. Scale bars = 200 μm in (A through D), Scale bars = 100 μm in (a through d). [file scrt228-S3.tiff]
